# Re-Tear Rates Following Rotator Cuff Repair Surgery

**DOI:** 10.7759/cureus.34426

**Published:** 2023-01-31

**Authors:** Jamie C Routledge, Ahmed Y Saber, Neil Pennington, Neha Gupta

**Affiliations:** 1 Trauma and Orthopedics, Calderdale and Huddersfield NHS Foundation Trust, Huddersfield, GBR; 2 Plastic Surgery, Pinderfields Hospital, Wakefield, GBR

**Keywords:** rotator cuff mri, rotator cuff re-tear, arthroscopic rotator cuff repair, rotator cuff tear management, rotator cuff tears

## Abstract

Aim

Re-tears following rotator cuff repair surgery are a common occurrence. Previous studies have identified several factors that have been shown to increase the risk of re-tears. The purpose of this study was to evaluate the rate of re-tear following primary rotator cuff repair and to identify the factors that may contribute to this rate.

Method

The authors performed a retrospective review, looking at rotator cuff repair surgeries performed between May 2017 and July 2019 performed in a hospital by three specialist surgeons. All methods of repair were included. All patients' medical data, including imaging and operation records, were reviewed.

Results

A total of 148 patients were identified. Ninety-three males and 55 females with a mean age of 58 years (range 33-79 years). Thirty-four patients (23%) had post-operative imaging with either magnetic resonance imaging or ultrasound, where it was found that 20 (14%) had a confirmed re-tear. Of these patients, nine went on to have further repair surgery. The average age of the re-tear patients was 59 (age range 39-73) and 55% were female. The majority of the re-tears were from chronic rotator cuff injuries. This paper did not identify any correlation between smoking status or diabetes mellitus and re-tear rates.

Conclusions

This study indicates that re-tear after rotator cuff repair surgery is a common complication. The majority of studies find increasing age to be the biggest risk factor; however, this was not the case in our study which found females in their 50s to have the highest rate of re-tear. Additional research is required to understand what factors can contribute towards rotator cuff re-rupture rates.

## Introduction

Rotator cuff tears are one of the most common injuries seen by orthopedic surgeons with many suggesting the incidence to be as high as one in five people [1]. Rotator cuff tears in elderly patients are typically the result of age-related deterioration as opposed to younger patients where tears are more likely to be caused by trauma [2].

Despite rotator cuff tears being able to be defined by certain characteristics, there is no clear consensus on the classification used for this injury [3]. The main characteristics that are often used to classify the tear include size, depth, and location. Due to the high prevalence of rotator cuff tears, repair surgery is one of the most widely performed orthopedic surgeries, with arthroscopic repair being the preferred method [4]. Rotator cuff repair can decrease pain and increase function, thus improving a patient’s quality of life. Studies have shown positive post-operative satisfaction from patients [5].

Tears in the rotator cuff can be treated in one of two ways: conservatively or surgically [6,7]. The surgical procedures that can be performed include subacromial decompression, arthroscopic rotator cuff repair, and rotator cuff debridement (arthroscopic or mini-open). It has been demonstrated that there is no significant difference in the clinical benefits achieved by either of the arthroscopic approaches to rotator cuff repair [8,9].

Despite the advancements in procedures for repair, re-tear of the repaired tendon is one of the most common complications encountered post-operatively [10]. Re-tear rates range between 13% and 94% of cases [11]. Some studies have suggested that patients who have a re-tear following repair surgery frequently still have a significant recovery in comparison with their preoperative state. Furthermore, the re-rupture is typically smaller than the original tear [12]. Studies have identified a number of risk variables that have been demonstrated to increase the likelihood of a rotator cuff re-tear occurring. These variables include increasing age, significant tear size, female gender, poor muscle quality, and thicker tears [12-16]. The purpose of this retrospective study was to evaluate the rate of re-rupture following primary rotator cuff repair and to identify the factors that may contribute to this.

## Materials and methods

For this review, authors identified 148 patients who had been admitted to either Huddersfield Royal Infirmary Hospital or Calderdale Royal Hospital from May 2017 to June 2019. Patients who had undergone surgical fixation for a rotator cuff repair were eligible for inclusion in this study. All arthroscopic repairs performed by our orthopedic surgeons specializing in the treatment of upper limbs were included. Patients who were managed with a conservative approach were excluded from the study.

This paper accounted for acute and chronic rotator cuff tears of any size. We classified any injury over three months as chronic. We did not enquire as to the mechanism of injury. Patients’ smoking status and whether they had diabetes mellitus were considered during the data collection. For the re-tear patients, where able, we classified the grade of their original rotator cuff tear using the Goutallier classification which is used for the assessment of muscle degeneration.

## Results

Of the 148 patients identified, our cohort consisted of 93 males and 55 females with a mean age of 58 years (range 33-79 years). The injuries were right-sided in 97 patients and left-sided in the remaining 51 patients.

Surgery to repair the rotator cuff was performed on 148 individuals. Arthroscopic rotator cuff repair was performed on 121 of these patients. Subacromial decompression was performed on 25 patients, and three individuals underwent both procedures in the operation. A total of 145 patients had a chronic tear in their rotator cuff, whereas three patients had an acute tear. Only 34 (23%) of the 148 patients who had arthroscopic surgery of their rotator cuff tear had a post-operative magnetic resonance image (MRI) performed. Following surgery, the average period of time prior to imaging being performed was 217 days.

**Figure 1:**
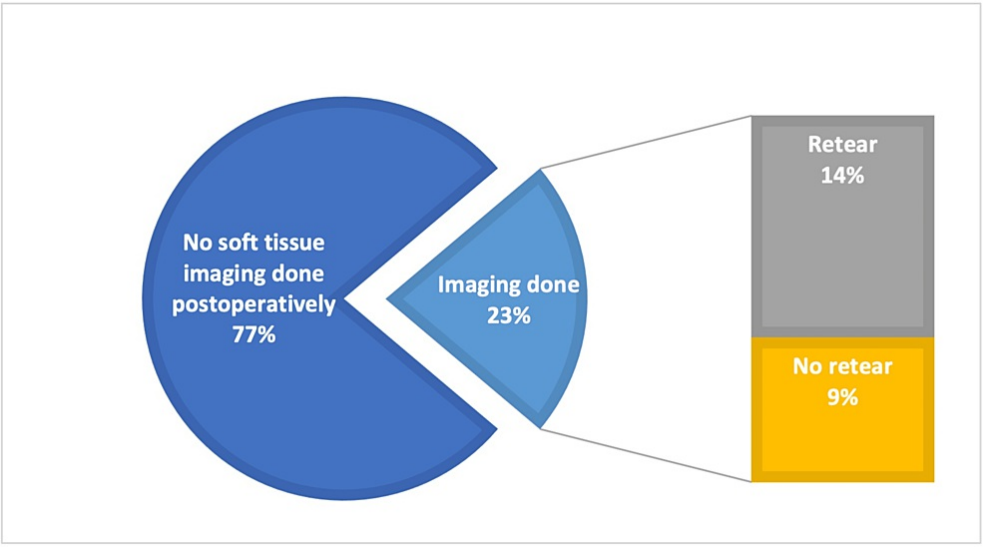
Graph to show our percentage of patients that had post-operative imaging

The data showed that MRIs and ultrasounds were performed on 34 patients with 27 undergoing MRI, six undergoing an ultrasound, and one patient receiving imaging from both modalities. The imaging showed that 14 (9%) patients did not have a re-tear, while 20 (14%) did (Figure 1). Of the 20 patients that had a confirmed re-tear, 18 (90%) had undergone arthroscopic repair whilst two (10%) had a subacromial decompression. Following the results of the imaging, nine out of the 20 patients who had a verified re-tear went on to have revision surgery. One patient did not wish to proceed with further surgery and the patients remaining who had a confirmed re-tear were conservatively managed.

Overall, of the 148 patients that were operated on, nine went on to receive a second repair surgery. The age range of these 20 patients who had a confirmed re-tear ranged from 39 to 73 years old, and 55% of them were female. Table 1 illustrates the re-tear rates in relation to the age groups of the patients. The median age of these patients was 59.65% of the re-tears on the right side, whilst the remaining 45% were on the left side.

**Table 1:** Rates of re-tear in relation to age from our study

Patients' age	Re-tear rates
Under 50	5%
50-59	40%
60-69	35%
79-79	20%
Over 80	0%

**Figure 2:**
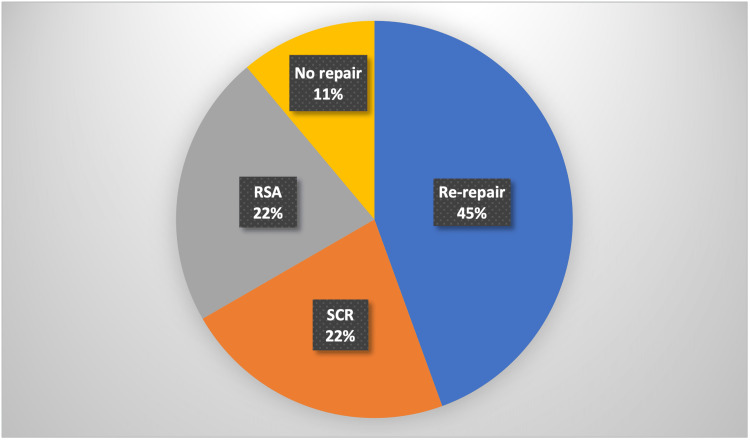
Repair methods used during further surgery RSA: reverse shoulder arthroplasty, SCR: superior capsule reconstruction

For the nine patients that went on to receive revision surgery, alternate operative methods were used. Four patients had a rotator cuff re-repair, two patients underwent a reverse shoulder arthroplasty (RSA) and two underwent a superior capsule reconstruction (SCR). Percentages are illustrated in Figure 2. One patient was found not to have a re-tear, therefore, no repair was needed. Out of the 20 patients who had a confirmed re-tear two were current smokers, two were previous smokers, and 16 were non-smokers. Two of the patients had a diagnosis of diabetes mellitus. Eighteen patients had chronic rotator cuff tears and only two had acute ones. Of the 20 patients who had a confirmed re-tear, 11 of these patients received pre-operative MRIs. We were then able to apply the Goutallier classification to grade their initial rotator cuff tear. Six patients had a grade zero, two had a grade one, one had a grade two, and two had a grade three.

A total of 115 patients had no confirmed or suspected rotator cuff re-tear. The majority of these patients were seen at least twice in the clinic post-operatively to monitor their progression and post-operative symptoms. During these consultations that were led by either a consultant or registrar, there were no concerns or suspicions of any complications including a re-tear. It was not uncommon for these patients to still have some degree of discomfort. However, it was felt by the clinicians that for these patients the degree of pain they were experiencing was to be expected for the stage that they were postoperative. Patients were discharged from the clinic up to one year following their rotator cuff repair if they had no post-operative concerns.

Despite a confirmed re-tear, 11 patients did not receive an operative repair and were instead managed conservatively. There were several factors influencing this decision. Firstly, each consultant’s individual view regarding operative vs. conservative management of re-tears played a role in the decision. Often the decision to manage conservatively was made in conjunction with offering alternatives for pain management such as shoulder injections or longer rehabilitation programs. Some patients simply did not want to have surgery and therefore declined an operation. For others, despite an MRI-proven re-tear, their range of movement had improved to a satisfactory level by the time the patients were seen in the clinic and therefore operative management was deemed not necessary. It has been shown in previous studies that conservative management should be trialed in low-demand patients [17].

## Discussion

Re-tear after a rotator cuff repair is a commonly encountered complication and has been shown to be caused by a combination of various factors. Previous studies have identified that the size of the rotator cuff tear and age are the greatest predictors of outcomes [18]. In addition, the quality of the tissue, limb dominance, and smoking status are all factors that can influence the likelihood of a re-tear occurring after the initial repair [19]. Although it has been shown that increasing age can increase the risk of re-tear, the number of re-tears that occurred in patients in their 50s was shown to be the highest of any age group [19,13]. Table 2 illustrates findings from other studies comparing re-tear rates and mean age.

**Table 2:** Comparison of our study with other studies Klepps et al. [20]; Lapner et al. [21]; Gallagher et al. [4]; Sheean et al. [22]; Ma et al. [23].

Cohort	Re-tear rate %	Mean age, year
Our study	13.5	59
Klepps et al.	31.3	64
Lapner et al.	27.6	56.8
Gallagher et al.	17.4	65.7
Sheean et al.	13.3	65
Ma et al.	30.2	61.2

In our research, we found that the female gender was associated with a higher prevalence of rotator cuff re-tears when compared to males. We found that 55% of those who had a re-tear were female, despite only 37% of the overall cohort of patients undergoing rotator cuff repairs being female. This varies from the findings of other studies that have shown that gender does not play a significant role in the development of rotator cuff injuries [24].

The management of re-tears in patients might vary based on a variety of criteria, such as the age of the patient, the patient's functional state, the magnitude of the re-tear, and the length of the tendon that is still intact. There are several different approaches that can be taken to address tears in the rotator cuff. These include conservative therapy, revision rotator cuff repairs, superior capsular reconstruction, tendon transfers, and reverse shoulder arthroplasty. Only 45% of patients in this study who had a rotator cuff re-tear confirmed on imaging proceeded to have surgical repair, while the remaining patients had conservative therapy instead.

The majority of patients are seen in the clinic post-operatively after the original rotator cuff repair surgery. During this consultation, following history and examination, the clinician decides whether to suspect a re-tear. There is often concern that a re-tear has developed if the patient has post-operative pain or decreased function following the repair [25]. In the post-operative reviews, both pain and reduced range of movement in the shoulder were all indications for the patient to have post-operative imaging. However, not all tears generate symptoms. Therefore, some patients may have tears that are not identified due to not receiving any form of imaging. MRI is the favored modality of imaging as although ultrasound is useful, it gives a limited comprehensive view of the shoulder [25]. The presence of atrophy and fatty infiltration on an MRI scan has been shown to correlate with failed rotator cuff repair. Failed repairs often show a progression of fatty infiltration and muscle atrophy [25].

In this study, postoperative imaging was found to be rather uncommon; with just 22% of patients receiving any form of imaging after their procedure. As a result, it is challenging to make an accurate assessment of the number of patients who sustained rotator cuff re-tears, as well as the potential causes of these tears and the mechanism through which they happened. The most common imaging modality chosen by clinicians to identify re-rupture rates was found to be MRI, with only a few clinicians choosing ultrasound to identify re-rupture rates [25]. When it comes to identifying partial thickness tears, several studies have demonstrated that ultrasound is highly reliable [26]. Studies have even shown it to be more specific than MRI. In these studies, it was found that ultrasound had a specificity of 66.7%, whilst MRI had a specificity of just 63.6% [27]. In light of the significant financial disparity between the two imaging modalities, it is essential to investigate the feasibility of performing ultrasounds for the diagnosis of re-rupture before resorting to MRI.

A limitation of our study is the lack of post-operative imaging that our patients received. As only 23% of the patients had post-operative imaging, we are unable to determine the true incidence of re-tear that may have occurred following the surgery.

## Conclusions

Our results suggest that arthroscopic rotator cuff repair is an effective surgery resulting in a positive outcome for the majority of patients. From our data, we can conclude the risk of a post-operative re-tear was 14%. However, we acknowledge not all patients had post-operative imaging. Our outcomes of rotator cuff repair appear consistent with the outcomes in the literature. Additional research is required to understand what factors can contribute towards rotator cuff re-rupture rates.
